# An Analytical Conductance Model for Gas Detection Based on a Zigzag Carbon Nanotube Sensor

**DOI:** 10.3390/s20020357

**Published:** 2020-01-08

**Authors:** Ali Hosseingholipourasl, Sharifah Hafizah Syed Ariffin, Mohammad Taghi Ahmadi, Seyed Saeid Rahimian Koloor, Michal Petrů, Afiq Hamzah

**Affiliations:** 1 UTM-MIMOS Center of Excellence in Telecommunication Technology, School of Electrical Engineering, Universiti Teknologi Malaysia, Skudai 81310, Johor, Malaysia; 2 Division of Computational Physics, Institute for Computational Science and Faculty of Electrical and Electronics Engineering, Ton Duc Thang University, Ho Chi Minh City 758307, Vietnam; 3 Institute for Nanomaterials, Advanced Technologies and Innovation, Technical University of Liberec, Studentska 2, 461 17 Liberec, Czech Republic; s.s.r.koloor@gmail.com (S.S.R.K.);; 4 School of Electrical Engineering, Universiti Teknologi Malaysia, Skudai 81310, Johor, Malaysia; mafiq@fke.utm.my

**Keywords:** analytical modeling, carbon nanotube, electrical conductance, field-effect transistor, gas sensor

## Abstract

Recent advances in nanotechnology have revealed the superiority of nanocarbon species such as carbon nanotubes over other conventional materials for gas sensing applications. In this work, analytical modeling of the semiconducting zigzag carbon nanotube field-effect transistor (ZCNT-FET) based sensor for the detection of gas molecules is demonstrated. We propose new analytical models to strongly simulate and investigate the physical and electrical behavior of the ZCNT sensor in the presence of various gas molecules (CO_2_, H_2_O, and CH_4_). Therefore, we start with the modeling of the energy band structure by acquiring the new energy dispersion relation for the ZCNT and introducing the gas adsorption effects to the band structure model. Then, the electrical conductance of the ZCNT is modeled and formulated while the gas adsorption effect is considered in the conductance model. The band structure analysis indicates that, the semiconducting ZCNT experiences band gap variation after the adsorption of the gases. Furthermore, the bandgap variation influences the conductance of the ZCNT and the results exhibit increments of the ZCNT conductance in the presence of target gases while the minimum conductance shifted upward around the neutrality point. Besides, the *I-V* characteristics of the sensor are extracted from the conductance model and its variations after adsorption of different gas molecules are monitored and investigated. To verify the accuracy of the proposed models, the conductance model is compared with previous experimental and modeling data and a good consensus is observed. It can be concluded that the proposed analytical models can successfully be applied to predict sensor behavior against different gas molecules.

## 1. Introduction

**Figure 1:**
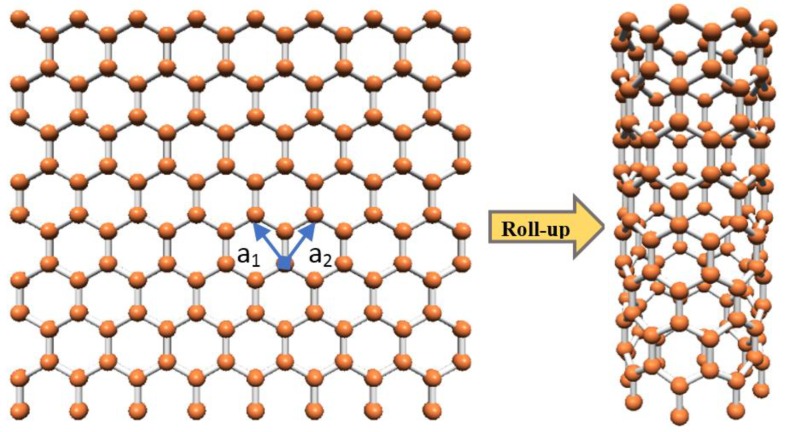
An SWZCNT model made up of the graphene layer.

The importance of environmental monitoring and protecting the human body from hazardous and toxic gases is a major motivation of the researchers to study and develop state of the art sensors with high sensitivity and ideal performance [1,2,3]. Gas sensors are used in various areas such as industrial, medical and household applications. The progress in nanoelectronics has proved that materials with smaller dimensions and the large surface area provide higher sensitivity and faster response times [4,5,6]. Among various nanomaterials, carbon nanotubes indicate a high potential for use in various applications such as transistors, capacitors, batteries and especially as a transducer in different types of gas sensors. As depicted in Figure 1, the carbon nanotube is a graphite sheet that is rolled up into a cylindrical shape that can be in the zigzag, armchair or chiral forms depending on the helicity of the tube [7].

In addition, based on the chiral angle and diameter, single-wall carbon nanotubes (SWCNTs) can be made to exhibit metallic or semiconducting properties being whether zigzag or armchair nanotubes [8,9,10,11]. These properties are determined by the geometrical parameter called chiral vector ${\vec{C}}_{h}=n{\vec{a}}_{1}+m{\vec{a}}_{2}$, where ${\vec{a}}_{1}$ and ${\vec{a}}_{2}$ are base vectors of the unit cell, and *n* and *m* are integers [12]. In the chiral vector, when the unit vectors *n* and *m* are equal, the CNT is an armchair type with metallic properties, and when *m* is zero the CNT is a zigzag nanotube that can be either metallic or semiconducting; else, it would be a chiral CNT [13,14]. The CNTs can also be distinguished from each other based on the edge shape at both ends of the tube, as ZCNT has zigzag shape and the armchair CNT has armchair shape. For the sensing applications, the semiconducting CNT is suggested to be more suitable than the metallic counterpart because metallic CNTs have a lower density of states around Fermi energy level compared to the semiconducting CNTs [15,16,17,18,19].

In recent years, gas sensors based on carbon nanotubes have been the subject of numerous studies for sensing and monitoring various gases of interest in different fields [20,21,22]. The required temperature for sensing various gases is different and it is important that sensors can operate at room temperature. Carbon nanotube-based gas sensors can recognize many toxic gases at room temperature [23]. On the other hand, the high surface area of the CNT sensors provides a fast response which may also cause slow recovery of these sensors, hence some techniques such as UV radiation can be used to improve the recovery time [23,24]. So far, sensors based on carbon nanotubes have been widely applied for the detection of a variety of hazardous gases such as CO, NH_3_, NO, NO_2_, etc. [25,26,27,28]. CNTs show superior performance because they have a high specific surface area that provides many sites for the gas molecules adsorption, and they have hollow geometry that aid to increase the sensitivity and decrease the operating temperature [29].

So far, many researchers have tried to fabricate, model and simulate gas sensors based on CNTs or other materials. Different methods and models have been developed and applied for modeling of gas sensors. For example, the modeling of CNT and graphene and GNR based sensors have been performed for the detection of NH_3_ and CO_2_ gas molecules by [30,31]. In the reported works, they considered gas concentration as a function of CNT and graphene and GNR conductance and tried to empirically formulate *I-V* variation after gas adsorption. Similar to this, another study was performed to detect NO_2_ gas using graphene-based FET platforms by [32]. They reported that NO_2_ concentration remains a factor that affects channel density of states, conductance and current-voltage characteristics. Furthermore, the gas concertation effect on the capacitance of the channel has been investigated by [33]. The capacitance has been considered as a function of CO_2_ gas concertation and using the relationship between current and capacitance, *I-V* characteristics in the presence of gas were derived. These studies have used completely similar approaches in the modeling process as they introduced some fitting parameters to fit the suggested models to experimental data. They did not provide physical and mathematical justification for fitting parameters used in the model. Therefore, the proposed models are neither analytical nor physical. In addition, the tight-binding (TB) approach has been adapted by various researchers for modeling nanostructure and nanosensors. For example, the TB approach was adapted to model graphene nanoribbon gas sensor to investigate the average density of states and the semiconducting energy gap of the GNR affected by the gas adsorption [34]. In addition, this technique has been used for modeling of GNR band structure and conductance [35], Boron nitride nanoribbons and SiC band structures modeling [36,37], modeling of gas effects on the local density of states of the GNR [38] and etc. In the reported works using the TB approach, they did not model nonequilibrium conditions and only statistical states have been analyzed that have been applied on graphene and GNR mostly. Other modeling techniques such as neural network model, Lasso and genetic algorithms or Wolkenstein adsorption theory and model have been used for modeling gas sensors based on carbon nanostructures [39,40]. However, these works did not investigate device physics and the sensing mechanism and the phenomena that occur inside the device, and just tried to interpret the output and formulate the output curves using algorithms.

Modeling of the nanosensors can guide the experiments by showing the possible behavior and how these behaviors depend on the methods that are chosen in simulation. In the sensing mechanism of the ZCNT, their physical and electrical properties are modified when exposed to the gas molecules. The strong molecular binding between the ZCNT surface and adsorbed gas molecules can exert some effects on the energy band structure of the ZCNT and alter its energy bandgap, thus, affect the conductance of ZCNT [38]. Also, the charge transfer induced from oxidizing or reducing adsorbed gas molecules on the ZCNT surface modulates its electrical resistance and affect the *I-V* characteristics [16,20,41]. In the case of oxidizing molecules adsorption and charge travel to the ZCNT surface, the concentration of charge carriers on the ZCNT increases and results in an increase in the conductivity of the channel [25].

**Figure 2:**
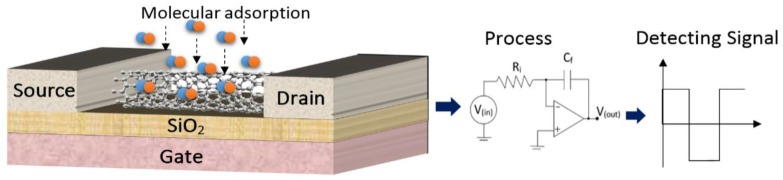
Illustration of a carbon nanotube FET based gas sensor.

Figure 2 shows a schematic of a single-wall carbon nanotube field-effect transistor-based gas sensor. The ZCNT act as a transducer in the sensor structure. The variation of electronic conductance as a result of charge travel to/from the ZCNT surface modifies the output current. The conductance and *I-V* characteristics of the ZCNT sensor can be implemented as the main sensing parameters to monitor and analyze the sensor performance.

## 2. Materials and Methods

**Figure 3:**
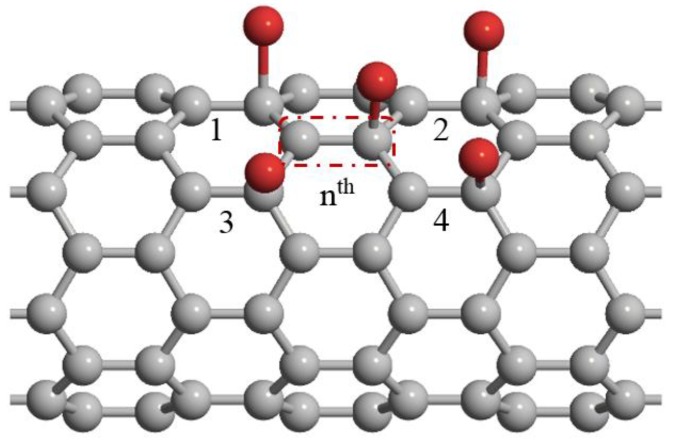
Configuration of adsorbed molecules on the ZCNT surface.

In this work, a ZCNT is assumed as a substrate and conducting channel connecting the source and drain electrodes. To model gas adsorption effects on the energy band structure of the ZCNT, the tight-binding (TB) technique is used. Now, let us consider a ZCNT that consists of *N* unit-cells. Each unit-cell is made of two carbon atoms. Here, we assume the adsorption of each gas molecule so that it is adsorbed to the second atom of the unit-cells as shown in Figure 3. We implement the nearest neighbor approximation in our TB model, so, for the *n^th^* unit cell, there are four neighbor unit cells. In the modeling of ZCNT energy band structure, the interaction of the carbon atoms was taken into account by hopping integral parameter. On the other hand, as discussed earlier, the electrical properties of carbon nanotubes strongly depend on their energy band structure. Based on the above concepts, we introduced another varying integral parameter and onsite energy parameter of the adsorbed molecule to address the interaction between gas molecules and carbon atoms and apply molecular adsorption effect on the ZCNT band structure. Definitely, this phenomenon will affect electrical properties such as conductance and current-voltage properties. Therefore, first, we start with modeling of the energy band structure by developing an energy dispersion relation with considering the molecular adsorption effect. Afterward, the conductance and *I-V* characteristics of the ZCNT are modeled through the energy dispersion relation.

### 2.1. Energy Band Structure Modeling

In order to model the energy band structure of the ZCNT considering the molecular adsorption effect, the Schrödinger equation [12] should be solved:

(1) Hψ(k,r)=E(k)ψ(k,r)

where *E* is the energy, *H* is the Hamiltonian operator matrix, *ψ* is the wave function and *k* is the propagation vector. Here we assumed plain wave as a wave function, thus:

(2) $$\psi =\psi_{0}e^{i\vec{k}.\vec{a}}\to \psi_{n}=\psi_{0}e^{i\vec{k}.{\vec{a}}_{n}}$$

Hamiltonian matrix operates on the wave vector and gives us the eigenvalue of the energy that we are looking for. Therefore, first, we have built the operator, which is a matrix. We are looking for the interaction between two adjacent carbon atoms. For simplicity, we assume that carbon atoms of the ZCNT have got only *s* orbital. Eventually, the Schrodinger equation can be written for the *nth* unit cell and its neighbor cells as:

(3) $$E_{n}\psi_{n}=\sum_{m=n,1,2,3,4}H_{nm}\psi_{m}=H_{n1}\psi_{1}+H_{n2}\psi_{2}+H_{n3}\psi_{3}+H_{n4}\psi_{4}+H_{nn}\psi_{n}$$

(4) $$h(\vec{k})=\left(\begin{array}{ccc} E_{0} & t & 0\\ t & E_{0} & t^{\prime }\\ 0 & t^{\prime } & E_{0}^{\prime } \end{array}\right)\times e^{ik\left(d-d\right)}+\left(\begin{array}{ccc} 0 & 0 & 0\\ t & 0 & 0\\ 0 & 0 & 0 \end{array}\right)\times e^{i\vec{k}a_{1}}+\left(\begin{array}{ccc} 0 & 0 & 0\\ t & 0 & 0\\ 0 & 0 & 0 \end{array}\right)\times e^{i\vec{k}a_{2}}+\left(\begin{array}{ccc} 0 & t & 0\\ 0 & 0 & 0\\ 0 & 0 & 0 \end{array}\right)\times e^{-i\vec{k}a_{1}}+\left(\begin{array}{ccc} 0 & t & 0\\ 0 & 0 & 0\\ 0 & 0 & 0 \end{array}\right)\times e^{-i\vec{k}a_{2}}$$

These matrix elements come from the interaction between the *nth* unit cell and its closest neighbors and adsorbed gas molecules. Here, *t* and *E*_0_ are the carbon-carbon hopping energy and onsite energy of the carbon atom, $t^{\prime }$ which is introduced as the hopping energy parameter between a carbon atom and an adsorbed gas molecule, *a*_1_ and *a*_2_ are the lattice vectors and $E_{0}^{\prime }$ is the on-site energy of the gas molecule. The achieved Schrödinger equation shows those neighbors (the nearest neighbors) that affect the *nth* unit cell and their energy.

On the other hand, in CNTs, the wave propagation parameters should satisfy the periodic boundary condition. Once the graphene sheet rolled up into a cylindrical shape, the value of the propagation parameter, *k*, is constrained by the imposition of periodic boundary condition along the chiral vector. The requirement of the periodic boundary condition for the ZCNT with a circumferential vector along the y-direction can be expressed as [12]:

(5) $$k_{y}=\frac{2\pi \upsilon }{2nb}$$

where *v* is the subband index, *n* is the chiral number, $b=\sqrt{3}a_{0}/2\dot{A}$, and $a_{0}=1.42\dot{A}$ which is nearest neighbor carbon to carbon atom distance in a graphene unit cell.

Finally, after the summation of the Hamiltonian matrixes over *n^th^* unit cell and four neighbor unit cells and calculating the determinant of the *h*(*k*) matrix, and applying the periodic boundary condition to the propagation parameter *k_y_*, the energy dispersion relation for the ZCNT is achieved:

(6) det(h(k)−EI)=0

(7) $$E_{zc}\left(\vec{k}\right)=\frac{1}{2}\left(\pm \sqrt{{\left(E_{0}+E_{0}^{\prime }\right)}^{2}-4E_{0}E_{0}^{\prime }+4{t^{\prime }}^{{}^{2}}+4t^{2}+16t^{2}\cos^{2}\left(\frac{\pi \upsilon }{n}\right)+16t^{2}\cos\left(\frac{\pi \upsilon }{n}\right)\cos\left(k_{x}a\right)}+(E_{0}+E_{0}^{\prime })\right)$$

where *I* is the identity matrix, $a=3a_{0}/2$ and *k_x_* is the wave propagation along the *x*-direction. Applying Taylor expansion to Equation (7), the final form of the energy dispersion relation is modified as:

(8) $$E_{zc}\left(\vec{k}\right)=\frac{1}{2}\left(\pm \sqrt{\alpha^{2}+\beta +16t^{2}\left(\gamma -\frac{k_{x}^{2}a^{2}}{2}+\frac{\pi^{2}\upsilon^{2}k_{x}^{2}a^{2}}{4n^{2}}\right)}+\alpha \right)$$

where $\alpha =E_{0}+E^{\prime }$, $\beta =4t^{2}+4{t^{\prime }}^{{}^{2}}-4E_{0}E_{0}^{\prime }$ and $\gamma =2+\frac{\pi^{4}\upsilon^{4}}{4n^{4}}-\frac{3\pi^{2}\upsilon^{2}}{2n^{2}}$ are introduced as coefficients.

In our proposed model in Equation (8), ${E^{\prime }}_{0}$ and $t^{\prime }$ are representing the molecular adsorption effects on the ZCNT band structure. It has been found that the hopping energy parameter, *t*, play a more important role in the physical and electrical properties than *E*_0_ [35,42,43]. Thus, in most calculations, the effect of *E_0_* has been neglected. Therefore, here the values of the *E*_0_ and $E_{0}^{\prime }$ are set to zero and we rely on $t^{\prime }$ to apply gas effects. However, this is important that all parameters be considered and appeared in our calculations. To apply the gas adsorption effect, the value of the $t^{\prime }$ for the adsorption of a specific gas on ZCNT can be calculated through [44]:

(9) $$t^{\prime }=t_{\alpha \beta }=t_{R}{\left(\frac{d_{R}}{d_{\alpha \beta }}\right)}^{2}$$

where *d_R_* stands for the distance between two adjacent carbon atoms of ZCNT, *t_R_* is the hopping integral between carbon atoms and $d_{\alpha \beta }$ stands for the bond length between ZCNT surface and adsorbed gas molecule. The distance and orientation of each gas are assumed according to the reported study by [45]. Thus, the value of the $t^{\prime }$ for the adsorption of each gas is presented in Table 1.

**Figure 4:**
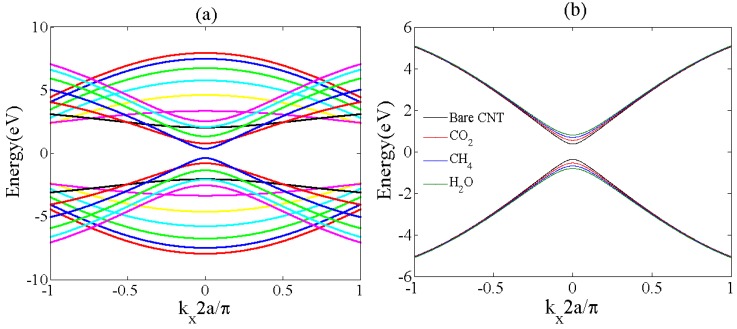
Band structure of ZCNT (13, 0) (**a**) bare ZCNT (**b**) ZCNT in after gas adsorption.

The electronic band structure of the ZCNT (13, 0) is illustrated in Figure 4. The zigzag CNT (13, 0) shows semiconducting properties, as there is a small gap between conduction and valence band around the Fermi energy. Furthermore, it can be seen that gas adsorption on the ZCNT surface influences on the energy band structure and modulates the energy bandgap. The gas adsorption on ZCNT and variation of the energy band gap lead to modification of the carrier concentration and modulate the electrical conductance and *I-V* characteristics of ZCNT which are modeled and discussed in the following section.

### 2.2. Conductance Model By Considering Gas Adsorption Effect

The variation of the energy band gap can significantly affect the conductivity of the ZCNT channel. The linear response conductance can be calculated through Landauer Formula is given as [46]:

(10) $$G=\frac{2q^{2}}{h}\int M\left(E\right)T\left(E\right)\left(-\frac{df}{dE}\right)dE$$

where *q* is the electron charge, *h* is the Plank’s constant and *f*(*E*) denotes the Fermi distribution function. The transmission probability, *T*(*E*), for the ballistic channel is equal to one [12]. The number sub-bands, *M*(*E*), depends on the location of sub-bands at a given energy. In the case that the energy includes the bottom of the conduction band, the parabolic approximation of the energy band diagram can be applied; then the number of sub-bands increases with energy. For the valence band, the sub-bands information cannot be explored easily as the number of coupled multiple bands is increasing and thus, a more complicated dispersion equation is required. Sub-bands density can be obtained by taking derivatives of the energy over the propagation vector:

(11) $$M\left(E\right)=\frac{\Delta E}{\Delta k\times L}$$

where *L* is the length of the ZCNT. Now Equation (11) can be modified as:

(12) $$M\left(E\right)=\pm \frac{\left(\frac{2t^{2}\pi^{2}\upsilon^{2}a^{2}k_{x}}{n^{2}}-4t^{2}a^{2}k_{x}\right)}{L\times {\left(\alpha^{2}+\beta +16t^{2}\left(\gamma -\frac{k_{x}^{2}a^{2}}{2}+\frac{\pi^{2}\upsilon^{2}k_{x}^{2}a^{2}}{4n^{2}}\right)\right)}^{\frac{1}{2}}}$$

while the wave propagation parameter in the parabolic part of the band structure is extracted as:

(13) $$k_{x}=\pm \sqrt{\frac{\left(2E^{2}-2\alpha^{2}-\beta -16t^{2}\gamma \right)n^{2}}{4\pi^{2}\upsilon^{2}a^{2}t^{2}-8a^{2}t^{2}n^{2}}}$$

Therefore, the general mathematical model for ZCNT can be written as:

(14) $$G=\pm \frac{2q^{2}}{h}\int \frac{\left(\frac{2t^{2}\pi^{2}\upsilon^{2}a^{2}k_{x}}{n^{2}}-4t^{2}a^{2}k_{x}\right)}{L\times {\left(\alpha^{2}+\beta +16t^{2}\left(\gamma -\frac{k_{x}^{2}a^{2}}{2}+\frac{\pi^{2}\upsilon^{2}k_{x}^{2}a^{2}}{4n^{2}}\right)\right)}^{\frac{1}{2}}}\times 1\times \left(-\frac{df}{dE}\right)dE$$

Finally, with the Fermi function $f(E)=1/\left(1+exp(\frac{E-E_{F}}{k_{B}T})\right)$ and normalized Fermi energy $\eta =E_{F}-E_{g}/k_{B}T$ and $x=E-E_{g}/k_{B}T$, the conductance model for the ZCNT channel with introducing the gas adsorption effect is formulated as:

(15) $$G=\pm \frac{2q^{2}}{h}\left(\frac{4t^{2}a^{2}n^{2}-2t^{2}\pi^{2}\upsilon^{2}a^{2}}{Ln^{2}}\right)\int \frac{k_{x}}{{\left(\alpha^{2}+\beta +16t^{2}\left(\gamma -\frac{k_{x}^{2}a^{2}}{2}+\frac{\pi^{2}\upsilon^{2}k_{x}^{2}a^{2}}{4n^{2}}\right)\right)}^{\frac{1}{2}}}\times \left(\frac{d}{dx}\left(\frac{1}{1+exp\left(x-\eta \right)}\right)\right)dx$$

**Figure 5:**
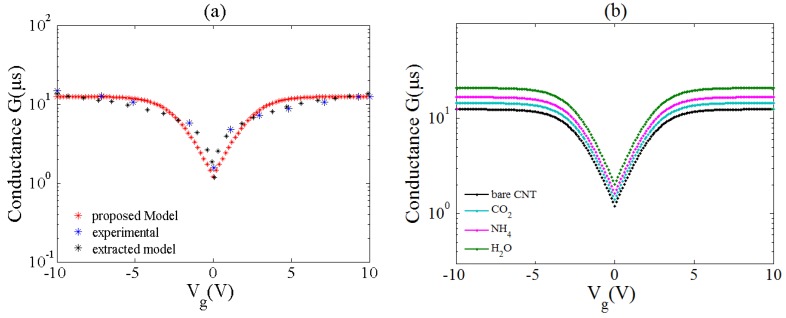
(**a**) Comparison of the proposed conductance model with experimental data and other modeling work. Acceptable consensus between our model and previous data can be seen; (**b**) Variation of the conductance in the presence of different gas molecules.

The conductance of the bare ZCNT is plotted respect to gate voltage in Figure 5a. The minimum conductance can be observed in charge neutrality point indicating higher resistance around this point. The comparison of the proposed model with other experimental and theoretical studies indicates a good agreement between the presented model in this work with previous studies [47,48]. Furthermore, according to the graph, the results of both modeling works are very close to the experimental work with negligible differences.

In addition, the other modeling work did not consider molecular adsorption effect and is developed for bare CNTFET. But, in our model, the sensing parameters are included to exert the gas adsorption effects. Thus, these parameters caused some differences between our model and reported works. In real applications, the conductance parameter is considered as a sensing parameter so that, they record conductance of the source-drain contacts before gas adsorption and measure its changes in the presence of gas molecules. By any changes, we can find out whether or not a gas molecule is adsorbed.

With $V_{g}=\frac{\eta k_{B}T}{q}+V_{T}$, the gate voltage can be related to the conductance relation. Here, *V_g_* is the gate voltage and *V_T_* is the thermal voltage. Figure 5b illustrates the variation of the ZCNT conductance after the adsorption of various gas molecules. It can be seen that after gas adsorption conductance of the ZCNT rises. This is because of the increase of the charge carrier density in the ZCNT surface that leads to an increase in the conductivity of the channel. In other words, in the adsorption process of the gas molecules as a result of the covalent bond created between target molecules and ZCNT, electrons be released and injected to the ZCNT surface that increases the carrier concentration. The conductance variation of the ZCNT channel directly modulates the *I-V* characteristics of the sensor. Assuming that the source and substrate potentials are grounded, the ZCNT channel exhibits resistor characteristics in small voltages of the Source-Drain (*V_DS_*). In addition, the general conductance model of the ZCNT can be implemented to describe the relationship between current and conductance. Therefore, based on the *I-V* characteristics of the ZCNT-FET based devices, the performance of the gas sensor can be evaluated through Equation (16):

(16) $$I_{D}=\left[\frac{2q^{2}}{h}\left(\frac{4t^{2}a^{2}n^{2}-2t^{2}\pi^{2}\upsilon^{2}a^{2}}{Ln^{2}}\right)\int \frac{k_{x}\left(\frac{exp\left(x+\eta \right)}{exp\left(x\right)-exp\left(\eta \right)}\right)}{{\left(\alpha^{2}+\beta +16t^{2}\left(\gamma -\frac{k_{x}^{2}a^{2}}{2}+\frac{\pi^{2}\upsilon^{2}k_{x}^{2}a^{2}}{4n^{2}}\right)\right)}^{\frac{1}{2}}}dx\right]\times V_{DS}$$

**Figure 6:**
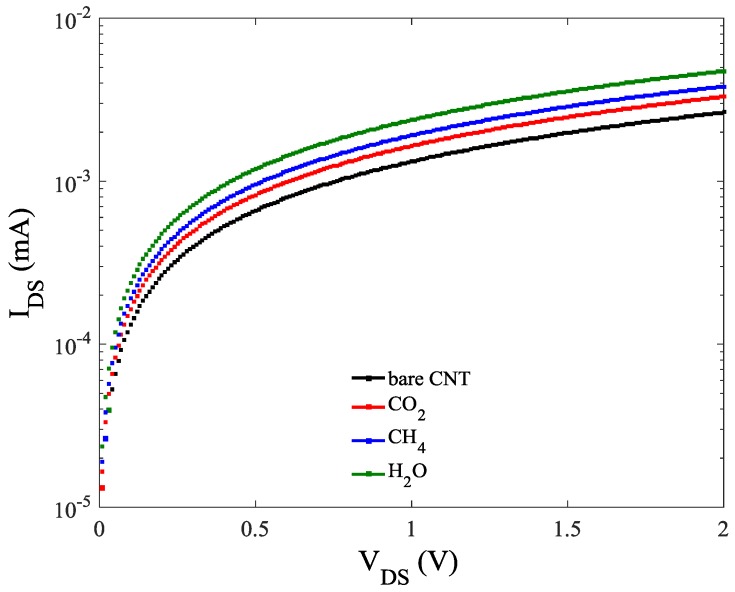
*I-V* characteristics of the gas sensor against different gas molecules.

The *I-V* characteristics of the ZCNT-FET based gas sensor before and after exposure to the gas molecules is presented in Figure 6 while the gate voltage is fixed at 1eV. It can be seen that current has raised after gas adsorption. In addition, the most and the least current increments are related to the H_2_O and CO_2_ adsorption. This means that in the process of H_2_O adsorption more electrons be injected to the channel and cause the highest electron concentration, thus the highest conductivity compared to the other two gases as depicted in Figure 6.

**Figure 7:**
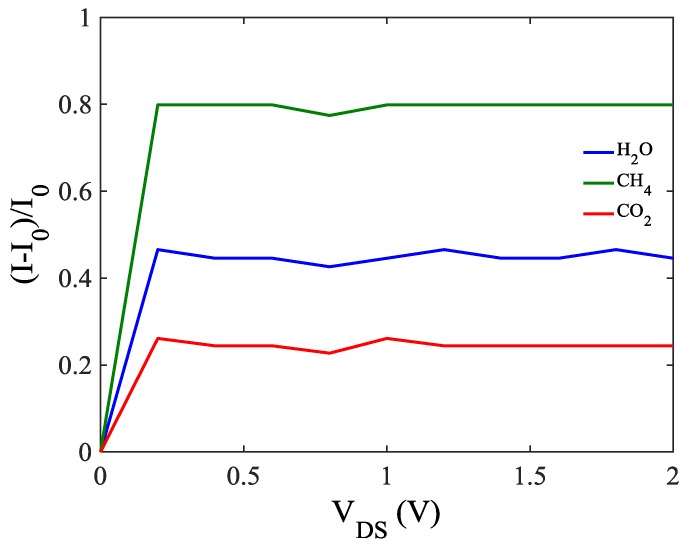
The response of ZCNT-FET based gas sensor toward adsorbed gas molecules.

Finally, to find out the sensitivity of the sensor, the sensor response analysis is performed as shown in Figure 7. The *I*_0_ and *I* are the currents before and after adsorption respectively. It can be seen that the sensor has almost high sensitivity especially at low voltages while it has a very sharp slope and become constant for the higher voltages. Furthermore, the sensitivity of the sensor against different gases can be understood and compared easily.

## 3. Conclusions

Gas sensors are being extensively used in diverse industries to monitor and control the leakage and concentration of gases and for safety purposes. Recently, a new generation of gas sensors based on carbon allotropes, especially carbon nanotubes, presenting excellent sensing performance has emerged. Therefore, in this paper, we propose a set of new analytical models to investigate the performance of the zigzag CNTFET-based gas sensor and analyze the variation of its physical and electrical properties against various target gas molecules. We used the tight-binding technique to build energy dispersion relation of ZCNT and to interpret the behavior of its electronic band structure after gas adsorption. Through this, conductance and *I-V* characteristics of the ZCNT sensor were formulated and applied for gas detection. On the other hand, a sensor response analysis was performed to measure semiconducting ZCNT against CO_2_, H_2_O, and CH_4_ gases. To validate our calculations and mathematical models, a comparison study between our proposed conductance model and previously published experimental data was performed and an acceptable agreement was achieved. It can be concluded that our proposed models can be applied to study various properties of the carbon nanotubes to fabricate modern sensors with a strong performance.

## Figures and Tables

**Table 1:** The calculated hopping energies for adsorbed gas molecules.

Adsorption Type	Distance from ZCNT Surface (Å)	Hopping Parameter
CH_4_	$d_{\alpha \beta }=3.19$	t_C-CH4_ = 0.445t_R_
CO_2_	$d_{\alpha \beta }=3.23$	t_C-CO2_ = 0.43t_R_
H_2_O	$d_{\alpha \beta }=2.69$	t_C-H2O_ = 0.528t_R_
